# Keratoconus severity identification using unsupervised machine learning

**DOI:** 10.1371/journal.pone.0205998

**Published:** 2018-11-06

**Authors:** Siamak Yousefi, Ebrahim Yousefi, Hidenori Takahashi, Takahiko Hayashi, Hironobu Tampo, Satoru Inoda, Yusuke Arai, Penny Asbell

**Affiliations:** 1 Department of Ophthalmology, University of Tennessee Health Science Center, Memphis, Tennessee, United States of America; 2 Department of Genetics, Genomics, and Informatics, University of Tennessee Health Science Center, Memphis, Tennessee, United States of America; 3 Department of Ophthalmology, Jichi Medical University, Tochigi, Japan; 4 Department of Ophthalmology, Yokohama Minami Kyosai Hospital, Kanagawa, Japan; Federal University of Technology - Paraná, BRAZIL

## Abstract

We developed an unsupervised machine learning algorithm and applied it to big corneal parameters to identify and monitor keratoconus stages. A big dataset of corneal swept source optical coherence tomography (OCT) images of 12,242 eyes acquired from SS-1000 CASIA OCT Imaging Systems in multiple centers across Japan was assembled. A total of 3,156 eyes with valid Ectasia Status Index (ESI) between zero and 100% were selected for the downstream analysis. Four hundred and twenty corneal topography, elevation, and pachymetry parameters (excluding ESI Keratoconus indices) were selected. The algorithm included three major steps. 1) Principal component analysis (PCA) was used to linearly reduce the dimensionality of the input data from 420 to eight significant principal components. 2) Manifold learning was used to further reducing the selected principal components nonlinearly to two eigen-parameters. 3) Finally, a density-based clustering was applied to the eigen-parameters to identify eyes with keratoconus. Visualization of clusters in 2-D space was used to validate the quality of learning subjectively and ESI was used to assess the accuracy of the identified clusters objectively. The proposed method identified four clusters; I: a cluster composed of mostly normal eyes (224 eyes with ESI equal to zero, 23 eyes with ESI between five and 29, and nine eyes with ESI greater than 29), II: a cluster composed of mostly healthy eyes and eyes with forme fruste keratoconus (1772 eyes with ESI equal to zero, 698 eyes with ESI between five and 29, and 117 eyes with ESI greater than 29), III: a cluster composed of mostly eyes with mild keratoconus stage (184 eyes with ESI greater than 29, 74 eyes with ESI between five and 29, and 6 eyes with ESI equal to zero), and IV: a cluster composed of eyes with mostly advanced keratoconus stage (80 eyes had ESI greater than 29 and 1 eye had ESI between five and 29). We found that keratoconus status and severity can be well identified using unsupervised machine learning algorithms along with linear and non-linear corneal data transformation. The proposed method can better identify and visualize the keratoconus stages.

## Introduction

Keratoconus is a noninflammatory ectatic corneal disorder characterized by progressive thinning resulting in corneal protrusion and decreased vision [1]. Moderate to advanced keratoconus cases are easily diagnosed due to the presence of classic retinoscopic and biomicroscopic signs. However, detecting subclinical keratoconus is challenging because initial manifestations of keratoconus may be unclear, requireing a more comprehensive analysis of corneal characteristics including topography, elevation, thickness, and biomechanical properties [2, 3]. Many methods have been suggested for identifying keratoconic eyes using corneal topography information. However, most of the methods rely on subjective analysis of topographical maps which can be biased by the observer [4].

Among objective approaches for keratoconus identification, machine learning analysis has gained a lot of attension. Smolek and Klyce proposed a neural network for keratoconus screening based on corneal topography indices [5]. Chastang et al. introduced a binary decision trees method based on corneal topography indices to identify clinically apparent keratoconus from normal cornea [6]. A similar aproach was used a few years later to identify keratoconus from normal corneas using corneal surface modeled with a seventh-order Zernike polynomial [7]. All these methods used only anterior topography characteristics of cornea. However, with the advancement of technology, posterior corneal curvature and pachymetric data were acquired and used to evaluate corneal characteristics [8]. Pinero et al. documented the corneal volume, pachymetry, and correlation of anterior and posterior corneal shape in subclinical and clinical keratoconus [9]. Perez et al. show that corneal instruments including videokeratography, Orbscan, and Pentacam together with the indices can lead to early keratoconus detection, however, with an increase in false positive detection [10].

Current methods for automatic detection of keratoconus are mainly supervised, in the sense that labels and diagnoses are required as input for subsequent machine learning. We propose an approach that is non-biased by either clinician or patient. This approach may lead to better identification of form fruste keratoconus which can be hard to do clinically in some cases. Moreover, it provides a non- biased method to determine progression and need for other treatment, such as cross-linking [11]. From big data perspecitve, the propsoed approach is objective without the need to pre-label the eyes. Our results suggest that unsupervised machine learning can be applied to corneal topography, elevation, and pachymetry parameters to generate highly specific and sensitive models.

## Material and methods

### Patients and instrument-guided screening index

In this multi-center retrospective study, we collected corneal optical coherence tomography (OCT) images from 12,242 eyes of 3162 subjects using SS-1000 CASIA OCT Imaging Systems (Tomey, Japan) and other parameters from the electronic health record (EHR) system. All data available at each instrument was collected without any pre-condition. We then selected a single visit from each eye and excluded eyes with missing Ectasia Status Index (ESI). A total of 3,156 eyes met the criterion. About 57% of the participants were female and the mean age was 69.7 (standard deviation; SD = 16.2) years. Three screening labels were derived from the ESI index of Casia; normal if ESI is between 0 and 4, forme fruste keratoconus (or keratoconus-suspect) if ESI is between 5 and 29, and keratoconus if ESI is 30 or greater. Using Casia labels, our dataset included 1970 healthy eyes, 796 eyes with forme fruste keratoconus, and 390 eyes with keratoconus. ESI is basically an instrument-guided screening index which has been shown to have a good agreement with Belin-Ambrósio (BA) index in diagnosing keratoconus [12]. This study was performed in accordance with the ethical standards in the Declaration of Helsinki and institutional review board (IRB) was submitted and approved by the “Jichi Medical University IRB Office”. Data use agreement was signed between centers in Japan and our institute to conduct the analysis. The data was de-identified in Japan before any further processing.

### Machine learning analysis

Four hundred and twenty parameters including axial, refractive, elevation, and pachymetry of both anterior and posterior surfaces of cornea were selected for the unsupervised machine learning analysis. All ESI-related parameters were excluded from the dataset. We first applied a principal component analysis (PCA) using prcomp function in the R package to the 420 selected corneal parameters. PCA uses a linear and orthogonal transformation to convert the observations of highly correlated corneal parameters into a set of new parameters which are linearly uncorrelated to each other. In another word, each new principal component parameter is a weighted combination of all initial corneal parameters while the components do not carry correlation anymore. This transformation allowed us to linearly reduce the number of dimensions of the original dataset. To investigate how many principal components are significant compared to a generated null distribution, we generated 100 independent artificial datasets such that within each dataset, the values along every corneal parameter were randomly permuted [13]. This operation removes the pairwise correlations between corneal parameters while keeping the distribution of every parameter unchanged. We then applied PCA to each of these 100 artificial VF datasets and sorted the combined eigenvalues of different datasets. We identified the principal components in our dataset in which their eigenvalues were significantly greater than the top eigenvalues from the artificial datasets (p < 0.01, Bonferroni corrected).

We then applied manifold learning using t-distributed stochastic neighbor embedding (tSNE) [14] to group eyes with similar corneal characteristics together and to separate eyes with dissimilar characteristics as far away as possible. We used Rtsne function in the R package for this purpose. This process maps eyes with similar local distance metrics in the tSNE space and nonlinearly reduce the dimension of input data. Moreover, tSNE is well-suited for visualization and monitoring the progress of the disease by clinicians since it provides a user-friendly visualization. Moreover, it allows subjective validation of the follow-up unsupervised clustering because one can see how the clusters are distributed and overlapped in 2-dimensional space. More importantly, tSNE generates more distinct and non-overlapping clusters compared to the best two principal components.

While there are several unsupervised clustering algorithms for identifying hidden structures in datasets [15–20], we employed an unsupervised density-based clustering [21] in the tSNE space to identify eyes with similar corneal characteristics in tSNE space and to group the eyes into non-overlapping clusters objectively. Density-based clustering groups eyes in the tSNE space that that are closely packed together and have many neighbors around them while eyes that lie alone (in low-density areas) and are too far away will be marked as outlies and not members of groups. We then assessed the accuracy of the approach both qualitatively (visualization) and quantitatively (using screening index of the Casia instrument).

## Results

**Fig 1:**
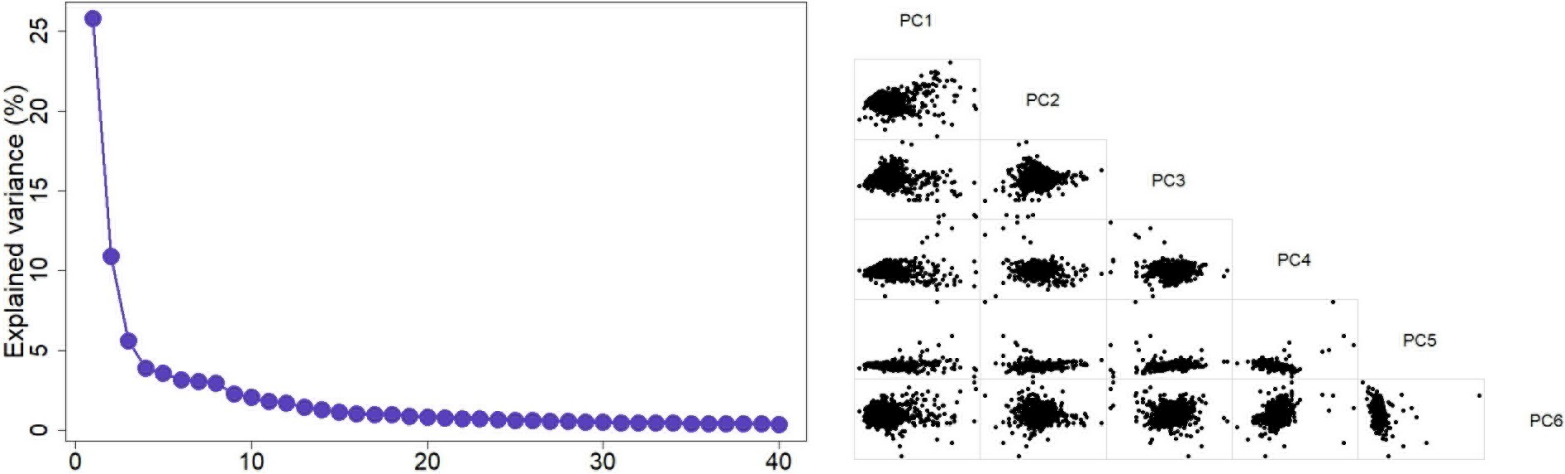
Applying principal component analysis on corneal features. **Left**: explained variance of the first 40 significant principal components. **Right**: corneal features in the space of the first six principal components.

**Fig 2:**
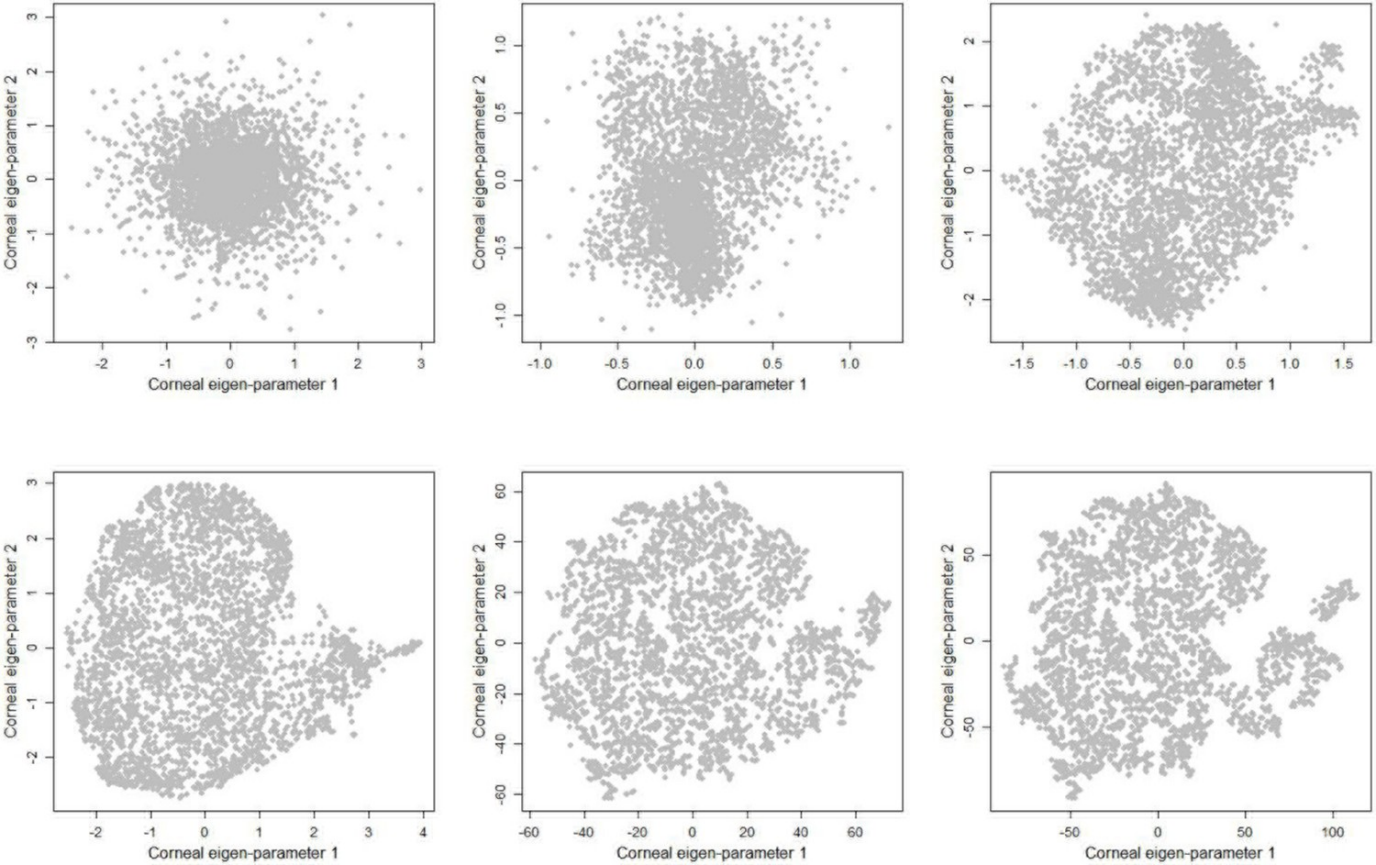
Evolution of corneal parameters in 2-D tSNE space. Zigzag, left to right, shows the evolution of tSNE over time starting from initial state which the corneal parameters are simply collapsed onto a 2-D space and then grouping eyes with similar corneal characteristics together over time.

**Fig 3:**
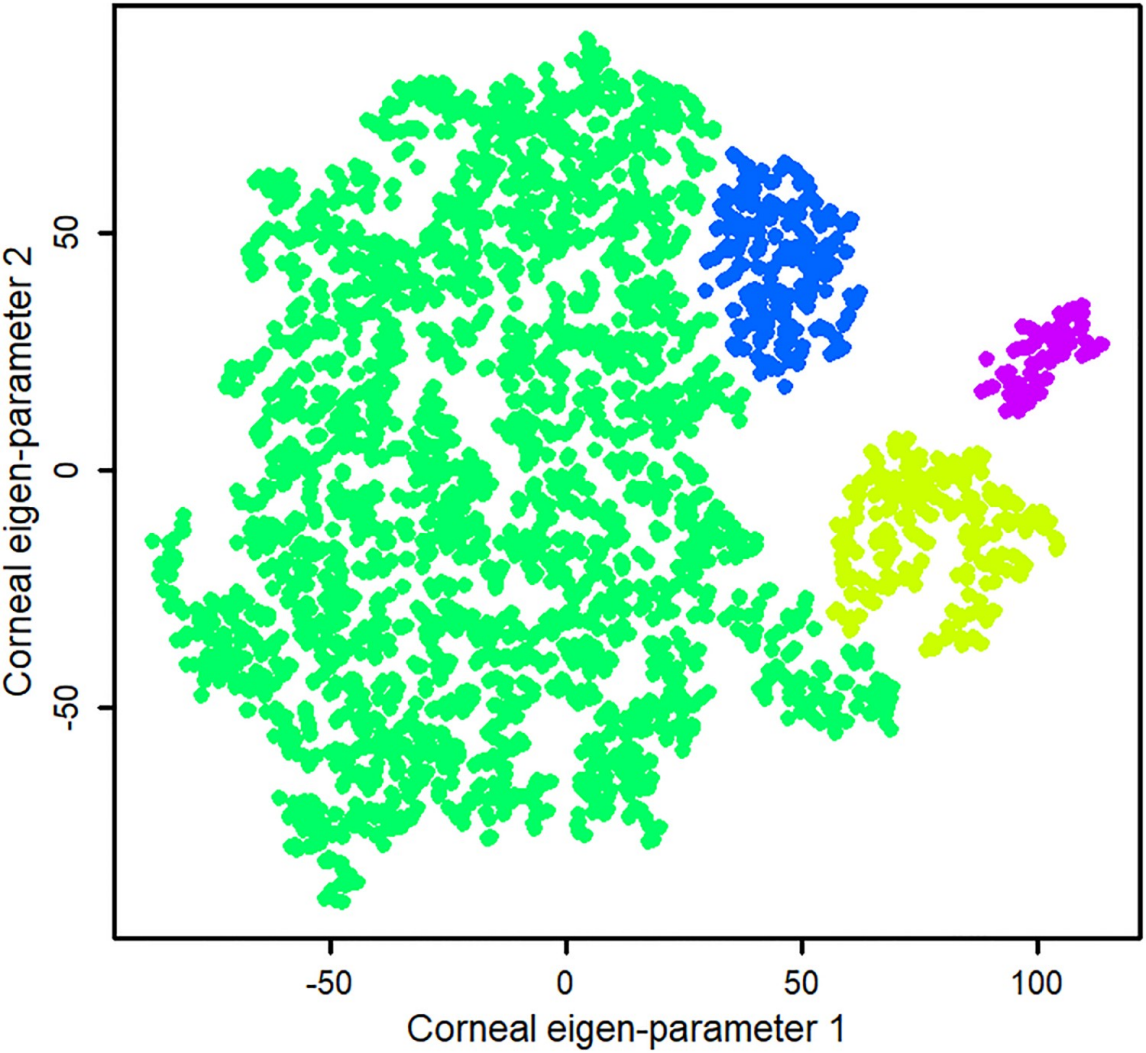
Unsupervised machine learning identified four clusters of eyes with similar corneal characteristics.

Fig 1 (left) shows the top 40 principal components and the amount of variance in data explained by those components. We identified 32 principal components as significant based on our quantitative analysis. The top 32 principal components explained over 80% of the total variability in the data while the top eight principal components carried approximately 60% of the total variability in the data. However, after investigating tSNE maps, we selected 8 principal components which showed a more discriminative clusters on the tSNE map (qualitative validation). We generated two corneal eigen-parameters which is essentially a nonlinear combination of original corneal parameters. Fig 2 shows the evolution of tSNE over time starting from the initial state in which the corneal parameters are collapsed in 2-D space without considering the local characteristics among points. We selected 2-D because it provides a user-friendly, importantly, a clinician-friendly visualization. The algorithm then identifies eyes with similar characteristics based on their distances in the tSNE space and gradually groups them together. The perplexity which reflects the assumed number for the neighbors for each point was set to 34 and we allowed the maximum number of iterations to 1000. To subjectively assess the accuracy of learning, we applied unsupervised density-based clustering on the two identified corneal eigen-parameters. Clusters with fewer than seven eyes were excluded. Unsupervised density-based clustering identified four non-overlapping clusters. For a better visualization, we color-coded the clusters as shown in Fig 3.

We then assigned clinical labels to the four identified clusters based on the ESI index (ranging from 0 to 100) provided by Casia instrument, where zero indicates normal and 100 reflects the most advanced stage of keratoconus. Casia instrument also provides diagnostic labels based on the ESI index: normal if ESI equals to zero, forme fruste keratoconus (or keratoconus-suspect) if ESI is between 5 and 29, and keratoconus if ESI is greater than 29. However, it is unclear how this index is generated from all corneal parameters and, more importantly, how the threshold for identifying eyes with forme fruste keratoconus is identified. Moreover, the currently used forme fruste keratoconus threshold index is confusing by its nature since keratoconus represents a spectrum of corneal deformations particularly in the early stages of the disease and it is challenging to assign a binary label to segregate a normal eye from an eye with forme fruste keratoconus. Nevertheless, using the Casia ESI index and diagnostic labeling convention, we determined that cluster I (color-coded blue) was mainly composed of healthy eyes: 224 healthy eyes, 23 eyes with forme fruste keratoconus, and nine eyes with keratoconus. Cluster II (color-coded green—big cluster on the left) was mainly composed of healthy eyes and eyes with forme fruste keratoconus: 1772 healthy eyes, 698 eyes with forme fruste keratoconus, and 117 eyes with keratoconus. Cluster III (color-coded light green) was mostly composed of eyes with mild keratoconus: 184 eyes with mild keratoconus, 74 eyes with forme fruste keratoconus, and six healthy eyes. The small cluster IV (color-coded purple) was mainly composed of eyes with advanced keratoconus: 80 eyes with advanced keratoconus and one eye with forme-fruste keratoconus.

**Fig 4:**
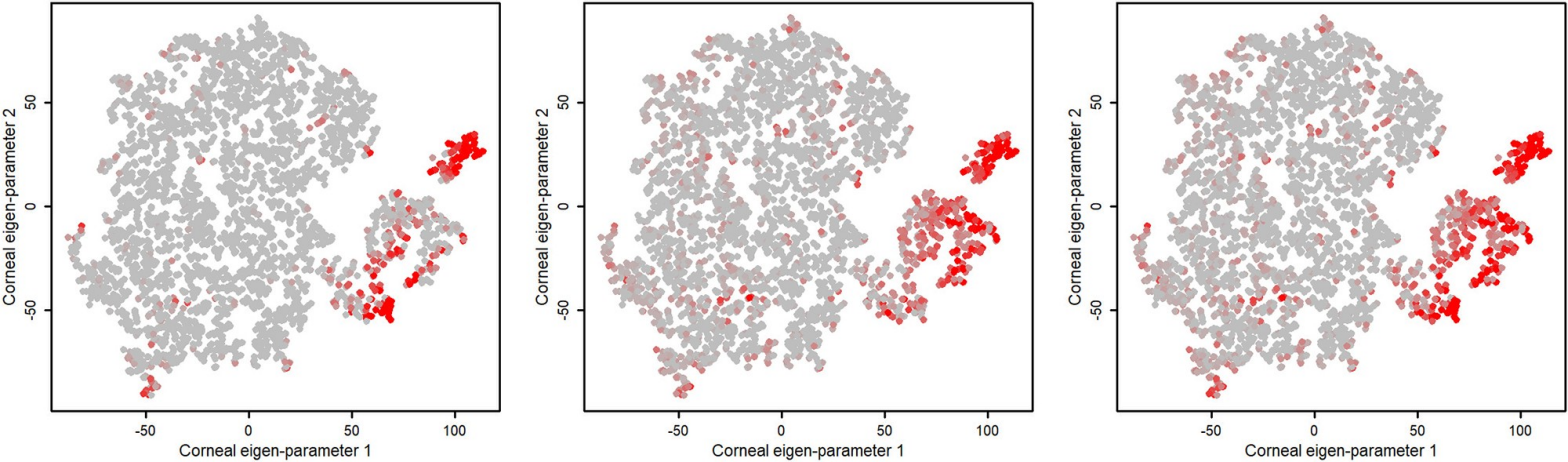
Mapping ESI index on clustering. **Left**: ESI index corresponding to anterior segment of cornea, **Middle**: ESI index corresponding to posterior segment of cornea, and **Right**: overall ESI index of Casia instrument.

To subjectively evaluate the correlation between the severity of keratoconus of eyes in the identified clusters and the ESI index of the Casia instrument, we color-coded each eye on the clustering plot with anterior, posterior, and total ESI indices reflecting the severity of keratoconus. Fig 4 shows the mapping of anterior, posterior, and total ESI indices onto the clusters we identified.

To objectively assess the accuracy of unsupervised clustering, we computed the specificity and sensitivity based on Casia diagnostic labeling. The specificity of identifying healthy eyes from eyes with keratoconus was 94.1% and the sensitivity of identifying eyes with keratoconus from healthy eyes was 97.7%.

**Fig 5:**
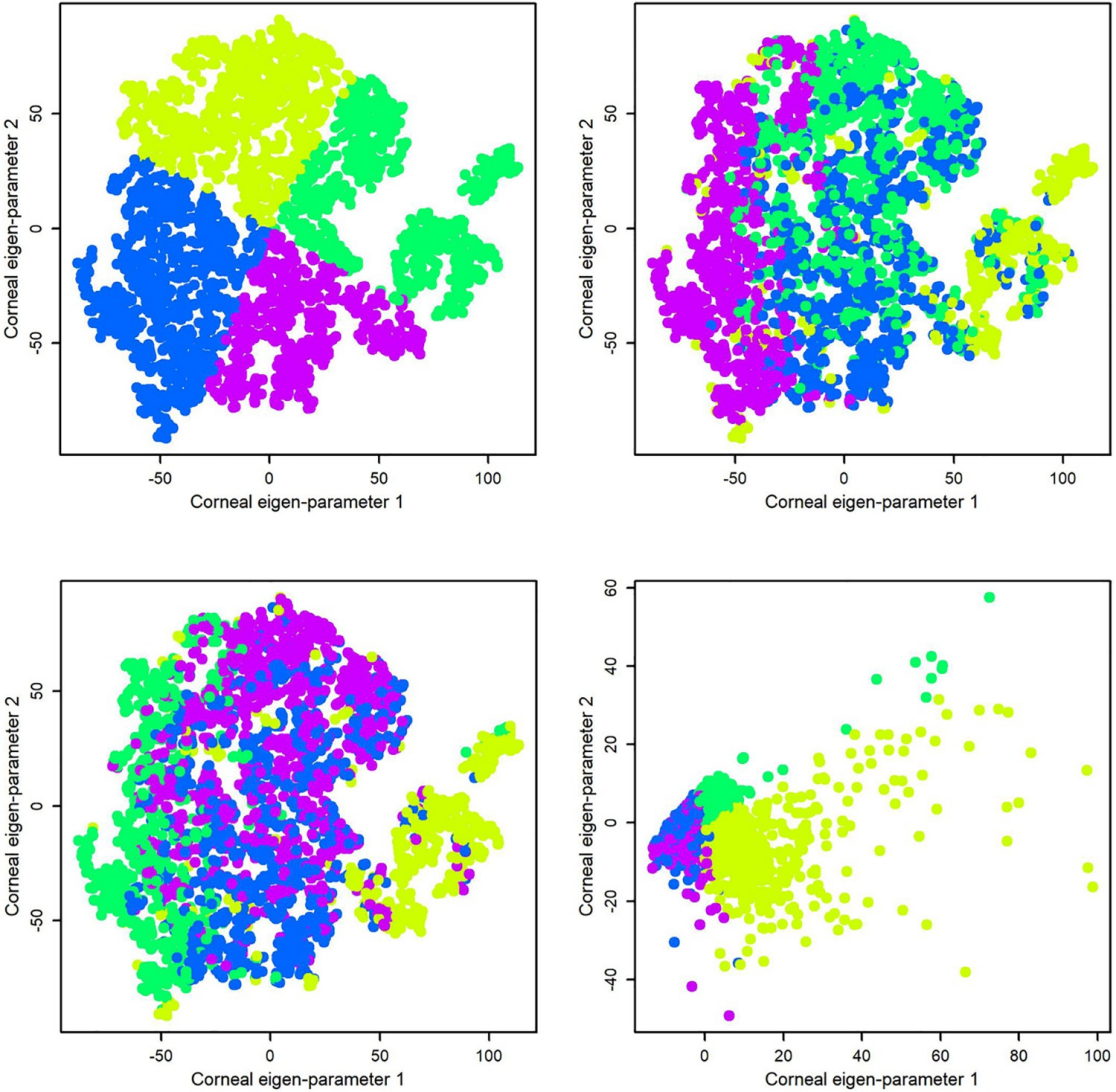
Investigating CLUTO, another density-based clustering algorithm. Top left: CLUTO was applied on the tSNE eigen-parameters and visualized on the tSNE map, Top right: CLUTO was applied on the PCA components and visualized on the tSNE map, Bottom left: CLUTO was applied on the original data with 420 parameters and visualized on the tSNE map, Bottom right: CLUTO was applied on the original data and visualized using two significant principal components.

To compare the DBSCAN clustering algorithm to other approaches, we investigated the OPTICS [19] and the Clustering Toolkit (CLUTO) algorithm [20]. CLUTO is a software package for unsupervised clustering of low- and high-dimensional datasets. We first applied CLUTO on the tSNE and visualized the outcome. We then asked whether CLUTO generates more discriminant clusters using principal components or the original data with 420 parameters. Fig 5 shows how CLUTO clustered the eyes using tSNE eigen-parameters, principal components, and original data. As can be seen, none of the outcomes generated a well-separated clusters. To assess the outcome of clustering objectively, we further investigated the specificity and sensitivity of the clusters using the same approach that we performed for DBSCAN. We determined that DNCLUE generates four clusters that are typically normal and one cluster that is abnormal. We used optics and skmeans functions in R to implement OPTICS and CLUTO, respectively.

To investigate the clusters generated by CLUTO algorithm objectively, we calculated the specificity and sensitivity of CLUTO applied to the original data with 420 parameters. The specificity of identifying healthy eyes from eyes with keratoconus was 97.4% and the sensitivity of identifying eyes with keratoconus from healthy eyes was 96.3%. However, we selected DBSCAN applied on tSNE since this combination provided an acceptable accuracy and well-separated clusters matched with different stages of keratoconus.

## Discussion

The major finding of our study is that automated, unsupervised clustering algorithms using topographic, tomographic, and thickness profiles of cornea provides a specific and sensitive means for determining keratoconus status and severity. The proposed unsupervised machine learning analysis for keratoconus diagnosis and staging provides a promising tool for improving the early detection of initial stages of keratoconus and for potential monitoring of treatment for the disease.

Marc Amsler first described how keratoconus manifests in altered corneal topography in 1938; however, the introduction of computer-aided videokeratoscopy in the early 1980’s revolutionized the diagnosis of keratoconus. Most of the early methods and severity indexes for identifying keratoconus have subsequently been based on corneal topography [2, 4, 9, 22–25]. More recently it was determined that pachymetric indices were better able to differentiate healthy eyes from eyes with keratoconus, based on a cohort of 44 eyes with keratoconus and 113 healthy eyes.[26] However, in the current study we used topography, elevation, and thickness profiles of corneal extracted from optical coherence tomography (OCT) images from subjects using the SS-1000 Casia to identify and stage keratoconus.

Historically, classification of the stages of keratoconus has been based on qualitative analysis of overall corneal morphology. However, we used machine learning because it addresses limitations of currently used diagnosis methods, including qualitative rather than quantitative parameter assessments and observer bias. While machine learning algorithms for keratoconus have been previously proposed, most are based on either a single type of corneal parameter (e.g., topography alone)[23–25] or require pre-labeled data [4, 7, 27]. For instance, some researchers have used supervised neural network or tree-based classification to discriminate between normal eyes and eyes with keratoconus [4, 27–29]. However, pre-labeling an eye as keratoconus or forme fruste keratoconus subjectively itself is prone to subjective evaluation and bias.

We used approximately 420 corneal parameters generated by Casia instrument through swept source OCT images of the cornea. All these corneal parameters were transformed to a 2-D space using linear PCA and non-linear tSNE followed by an unsupervised machine learning algorithm. Therefore, we first extract the information that is highly predictable of the corneal status instead of feeding all parameters to the machine learning and confuse its prediction. However, most of the machine learning algorithms in the literature simply input different corneal parameters to a machine learning algorithm to identify keratoconus without leveraging the power of data transformation and extracting most informative knowledge for identifying disease.

To investigate whether PCA alone is able to generate well-separated clusters comparable to those identified by the combination of the PCA and tSNE, we applied PCA alone and performed clustering. We found that PCA alone generated clusters with significant overlap. We also applied CLUTO on the selected principal component to compare the outcome with tSNE and observed similar overlapping clusters (Fig 5, top right). Subjective assessment of the quality of learning using visualization of the clusters and overlaying the ESI keratoconus index of the Casia (as shown in Fig 4) revealed that the ESI index of anterior corneal surface is highly correlated to the keratoconus severity of the eyes we identified in clusters. Specifically, the eyes in the Cluster IV (color coded purple) and classified as having advanced keratoconus by machine learning, have high agreement with anterior, posterior, and overall ESI indices since almost all eyes in this cluster have dark red color.

The same analogy holds for eyes in Cluster I (color coded blue) classified as normal, based on machine learning. However, for Cluster III (small cluster, color coded light green), which represents mild keratoconus based on machine learning, eyes generally have a worse posterior ESI index compared to their anterior ESI index (Fig 4, left and middle panels). This finding may suggest that posterior corneal parameters better identify keratoconus; however, this finding needs further investigation. We also hypothesize that eyes, labeled as normal by Casia ESI labeling system, falling in this cluster are likely “forme fruste” keratoconus candidates which need more attention from clinicians. Finally, Cluster II (color coded dark green) that represents healthy eyes and eyes suspect of keratoconus based on machine learning, is in strong agreement with all ESI indices except at the far right tail. The eyes in this region are not in a good agreement with anterior and overall ESI indices. We hypothesize that this region could be a either a separate cluster or part of cluster III that we were unable to identify based on current data and algorithms used. It is also possible that the eyes in this small region have other eye conditions along with mild keratoconus for which we did not have enough information to characterize. However, Fig 4 (middle panel) indicates that the posterior ESI index was more effective than anterior ESI index (Fig 4, left panel). In fact, a study conducted by the Ambrosio group shows that posterior features are superior to anterior features in identifying keratoconus [30].

To objectively assess the clinical labels we assigned to clusters with the severity of eyes in those clusters, we assessed the number of eyes with either large or small ESI. All eyes in Cluster IV (advanced keratoconus by machine learning) had ESI index greater than 38. In this group 71 (out of 81) eyes had ESI greater than 60, which indicates advanced stages of keratoconus. For Cluster I (normal by machine learning), only seven eyes (out of 256) had an ESI index greater than 30, indicating that the overwhelming majority of eyes in this cluster were normal. Therefore, our clustering is in good agreement with ESI at the two sides of spectrum. Based on Casia ESI diagnosis labels, the specificity of our machine learning method in identifying normal from keratoconus eyes was 94.1% and the sensitivity of identifying keratoconus from normal eyes was 97.7%, considering only normal and advanced keratoconus clusters.

There are a number of limitations to our study which could be addressed in follow-up studies. We compared the clustering outcome with Casia ESI index and showed that there is a good agreement between our finding and ESI index spectrum (Figs 3 and 4). However, to assess the generalizability of this unsupervised clustering approach method, it needs to be validated by other keratoconus indices such as Bellin-Ambrosio (BA) index. Therefore, it is required to conduct another study to confirm how this approach is generalizable to corneal parameters generated by Pentacam instrument by accessing such datasets. Also, the accuracy of this approach can be validated if the clinical diagnosis labels of all eyes were available. However, accessing clinical diagnosis labels for all eyes in such big datasets is a challenging and tedious task. Nevertheless, it is beneficial to assess the proposed approach in a follow-up study with a dataset that includes clinical diagnosis labels.

We performed a qualitative and quantitate assessment to determine whether PCA alone or other clustering approaches generate well-separated clusters. We found that the OPTICS density-based clustering approach was able to segregate eyes at different stages of keratoconus while the CLUTO unsupervised clustering approach generated overlapping clusters. However, the most important aspect of our proposed approach lies in the visualization property and the tSNE 2-D map. This is critical in practical clinical settings in which it is more appropriate to monitor the progression of the diseases on a 2-D map rather than proposing a black-box without 2-D visualization.

In summary, we proposed a possible solution to address shortcomings of current approaches in keratoconus diagnosis and monitoring, including observer bias in pre-defining diagnosis and limitations in the providing only a binary outcome that the eye belongs to either normal or disease group. The introduced unsupervised machine learning algorithm requires no pre-labeled data for training and can automatically identify the keratoconus status of a given eye based on comprehensive corneal parameters, including topography, elevation, and thickness profiles. More importantly, it provides visualization of the status of the eye compared to other eyes at different stages of keratoconus which was lack in supervised machine learning methods. To the best of our knowledge, this is the first attempt to develop a fully unsupervised algorithm for keratoconus identification and monitoring.

## Conclusion

Keratoconus status and severity can now be well identified using automated unsupervised clustering algorithms using topographic, tomographic, and thickness profiles of cornea. This approach can be used in corneal clinics and research settings to better diagnose, monitor changes and progression and improve our understanding of corneal changes in keratoconus.

## References

[1] Rabinowitz YS. Keratoconus. Surv Ophthalmol. 1998;42(4):297–319. . 9493273 10.1016/s0039-6257(97)00119-7

[2] de Sanctis U, Loiacono C, Richiardi L, Turco D, Mutani B, Grignolo FM. Sensitivity and specificity of posterior corneal elevation measured by Pentacam in discriminating keratoconus/subclinical keratoconus. Ophthalmology. 2008;115(9):1534–9. 10.1016/j.ophtha.2008.02.020 . 18405974

[3] Gordon-Shaag A, Millodot M, Ifrah R, Shneor E. Aberrations and topography in normal, keratoconus-suspect, and keratoconic eyes. Optom Vis Sci. 2012;89(4):411–8. 10.1097/OPX.0b013e318249d727 . 22311193

[4] Maeda N, Klyce SD, Smolek MK, Thompson HW. Automated keratoconus screening with corneal topography analysis. Invest Ophthalmol Vis Sci. 1994;35(6):2749–57. . 8188468

[5] Smolek MK, Klyce SD. Current keratoconus detection methods compared with a neural network approach. Invest Ophthalmol Vis Sci. 1997;38(11):2290–9. . 9344352

[6] Chastang PJ, Borderie VM, Carvajal-Gonzalez S, Rostene W, Laroche L. Automated keratoconus detection using the EyeSys videokeratoscope. J Cataract Refract Surg. 2000;26(5):675–83. . 10831896 10.1016/s0886-3350(00)00303-5

[7] Twa MD, Parthasarathy S, Roberts C, Mahmoud AM, Raasch TW, Bullimore MA. Automated decision tree classification of corneal shape. Optom Vis Sci. 2005;82(12):1038–46. . 16357645 10.1097/01.opx.0000192350.01045.6f PMC3073139

[8] Ambrosio R Jr., Alonso RS, Luz A, Coca Velarde LG. Corneal-thickness spatial profile and corneal-volume distribution: tomographic indices to detect keratoconus. J Cataract Refract Surg. 2006;32(11):1851–9. 10.1016/j.jcrs.2006.06.025 . 17081868

[9] Pinero DP, Alio JL, Aleson A, Escaf Vergara M, Miranda M. Corneal volume, pachymetry, and correlation of anterior and posterior corneal shape in subclinical and different stages of clinical keratoconus. J Cataract Refract Surg. 2010;36(5):814–25. 10.1016/j.jcrs.2009.11.012 . 20457375

[10] Fernandez Perez J, Valero Marcos A, Martinez Pena FJ. Early diagnosis of keratoconus: what difference is it making? Br J Ophthalmol. 2014;98(11):1465–6. 10.1136/bjophthalmol-2014-305120 . 24759873 PMC4215270

[11] Brown SE, Simmasalam R, Antonova N, Gadaria N, Asbell PA. Progression in keratoconus and the effect of corneal cross-linking on progression. Eye Contact Lens. 2014;40(6):331–8. 10.1097/ICL.0000000000000085 . 25320958

[12] Spira C, Grigoryan A, Szentmary N, Seitz B, Langenbucher A, Eppig T. [Comparison of the specificity and sensitivity of various instrument-guided keratoconus indices and classifiers]. Ophthalmologe. 2015;112(4):353–8. Epub 2015/01/23. . 25609499 10.1007/s00347-014-3135-8

[13] Shalek AK, Satija R, Adiconis X, Gertner RS, Gaublomme JT, Raychowdhury R, et al Single-cell transcriptomics reveals bimodality in expression and splicing in immune cells. Nature. 2013;498(7453):236–40. 10.1038/nature12172 . 23685454 PMC3683364

[14] Laurens van der Maaten GH. Visualizing Data using t-SNE. Journal of Machine Learning Research. 2008;9:2579–605.

[15] Rocha LM, Cappabianco FAM, Falcão AX. Data clustering as an optimum-path forest problem with applications in image analysis. International Journal of Imaging Systems and Technology. 2009;19(2):50–68. 10.1002/ima.20191

[16] Rehioui H, Idrissi A, Abourezq M, Zegrari F. DENCLUE-IM: A New Approach for Big Data Clustering. Procedia Computer Science. 2016;83:560–7. 10.1016/j.procs.2016.04.265.

[17] Yousefi S, Balasubramanian M, Goldbaum MH, Medeiros FA, Zangwill LM, Weinreb RN, et al Unsupervised Gaussian Mixture-Model With Expectation Maximization for Detecting Glaucomatous Progression in Standard Automated Perimetry Visual Fields. Transl Vis Sci Technol. 2016;5(3):2 10.1167/tvst.5.3.2 . 27152250 PMC4855479

[18] Yousefi S, Goldbaum MH, Balasubramanian M, Medeiros FA, Zangwill LM, Liebmann JM, et al Learning from data: recognizing glaucomatous defect patterns and detecting progression from visual field measurements. IEEE Trans Biomed Eng. 2014;61(7):2112–24. 10.1109/TBME.2014.2314714 . 24710816 PMC4254715

[19] Ankerst M, Breunig MM, Kriegel H-P, #246, Sander r. OPTICS: ordering points to identify the clustering structure. Proceedings of the 1999 ACM SIGMOD international conference on Management of data; Philadelphia, Pennsylvania, USA. 304187: ACM; 1999. p. 49–60.

[20] Zhao Y, Karypis G, Fayyad U. Hierarchical Clustering Algorithms for Document Datasets. Data Min Knowl Discov. 2005;10(2):141–68. 10.1007/s10618-005-0361-3

[21] Martin Ester H-PK, Jörg Sander, Xiaowei Xu. A density-based algorithm for discovering clusters in large spatial databases with noise Knowledge discovery and data mining (KDD). 1996:226–31.

[22] Li X, Yang H, Rabinowitz YS. Keratoconus: classification scheme based on videokeratography and clinical signs. J Cataract Refract Surg. 2009;35(9):1597–603. 10.1016/j.jcrs.2009.03.050 . 19683159 PMC3712873

[23] Saad A, Gatinel D. Topographic and tomographic properties of forme fruste keratoconus corneas. Invest Ophthalmol Vis Sci. 2010;51(11):5546–55. 10.1167/iovs.10-5369 . 20554609

[24] Tomidokoro A, Oshika T, Amano S, Higaki S, Maeda N, Miyata K. Changes in anterior and posterior corneal curvatures in keratoconus. Ophthalmology. 2000;107(7):1328–32. . 10889107 10.1016/s0161-6420(00)00159-7

[25] Holladay JT. Keratoconus detection using corneal topography. J Refract Surg. 2009;25(10 Suppl):S958–62. 10.3928/1081597X-20090915-11 . 19848378

[26] Ambrosio R Jr., Caiado AL, Guerra FP, Louzada R, Sinha RA, Luz A, et al Novel pachymetric parameters based on corneal tomography for diagnosing keratoconus. J Refract Surg. 2011;27(10):753–8. 10.3928/1081597X-20110721-01 . 21800785

[27] Carvalho LA. Preliminary results of neural networks and zernike polynomials for classification of videokeratography maps. Optom Vis Sci. 2005;82(2):151–8. . 15711463 10.1097/01.opx.0000153193.41554.a1

[28] Arbelaez MC, Versaci F, Vestri G, Barboni P, Savini G. Use of a support vector machine for keratoconus and subclinical keratoconus detection by topographic and tomographic data. Ophthalmology. 2012;119(11):2231–8. 10.1016/j.ophtha.2012.06.005 . 22892148

[29] Smadja D, Touboul D, Cohen A, Doveh E, Santhiago MR, Mello GR, et al Detection of subclinical keratoconus using an automated decision tree classification. Am J Ophthalmol. 2013;156(2):237–46 e1. 10.1016/j.ajo.2013.03.034 . 23746611

[30] Correia FF, Ramos I, Lopes B, Salomão MQ, Luz A, Correa RO, et al Topometric and Tomographic Indices for the Diagnosis of Keratoconus. International Journal of Keratoconus and Ectatic Corneal Diseases. 2012;1(2):92–9.

